# Liver-Assisting Devices

**Published:** 2009-12

**Authors:** Pramila Bajaj

**Affiliations:** Editor, IJA

Hepatic failure leads to a well-recognised pattern of clinical signs and symptoms; there is no specific therapy and the mortality rate is high (up to 80%)^1^. It results from severe impairment and / or necrosis of liver cells, which ultimately triggers multi-organ failure. Haemodynamic instability, renal failure, coagulopathy, profound metabolic disturbances and susceptibility to infections are common clinical features.

Hepatic encephalopathy with cerebral oedema is the key event that defines the prognosis, since death can occur even when the liver has begun to recover. Even though spontaneous liver regeneration is sometimes possible, it is, however, unpredictable; therefore, liver transplantation is the only therapeutic intervention of proven benefit.

Artificial hepatic support should provide metabolic, synthetic and detoxification functions, allowing time for recovery and regeneration of the host organ or for transplantation. Various liver-assisting therapies have been introduced since the early 1960s, but none has yet led to a significant clinical improvement, since it is still impossible to reproduce the unique and complex architecture of liver.

Basically, extracorporeal liver support systems are divided into biological, nonbiological (or artificial) and bioartificial (hybrid technique) devices.

**Biological methods:** with these approaches liver support-detoxification was achieved by whole portal and artery perfusion through animal or human livers. They are no longer applied.

**Artificial devices:** the main aim of these is to detoxify the patient by means of dialysis-derived techniques: plasma exchange, haemofiltration, haemodialysis (HD), albumin-dialysis, plasma adsorption, the Prometheus system.

Two of the most widely applied are:

MARS (molecular absorbent recirculating system), which is based on the principles of dialysis, filtration and adsorption. The patient's blood is brought into contact with a albumin-coated membrane which is capable of removing some toxins such as ammonia, bilirubin and aromatic aminoacids. The membrane adsorbs and holds the toxins for a time, but they are then released (following their concentration gradient) and are carried to the other side of the membrane, where dialysis against the albumin-rich dialysate removes the toxins from the membrane^2^.

PAP (plasma adsorption perfusion) is another nonbiological device by which plasma is first separated from blood and then passed through a filter where toxins (especially bilirubin) are adsorbed.

The artificial support systems are useful mainly for detoxification of some substances (ammonia, aromatic aminoacids, and bilirubin) and water-soluble toxins. Improvement of systemic haemodynamics and reduction of cerebral oedema and ICP have been demonstrated, but nothing has been reported relating to the promotion of liver synthetic function. Evidence of any benefit on survival is still lacking, since the removal of toxins, mediators, cytokines and other pro-inflamatory factors can be associated with the simultaneous removal of regenerating growth factors.

**Bioartificial devices:** with this approach biological tissues are combined with nonbiological materials; the aims of these devices are to provide both excretory and biotransformational functions and to remove cytokines and other toxins. The patient's blood passes through columns containing cultured hepatocytes (porcine cells for the BAL(bioartificial liver) and human hepatoblastoma cells for the ELAD(extracorporeal liver-Assisting device]).

While the bioartificial system have yielded real advantages in terms of neurological function and detoxification, serious drawbacks have nonetheless consistently limited their adoption, such as the risk of porcine retrovirus infections, graft-versus-host reactions, complement activation, activation of the clotting cascade, thrombocytopoenia, drug-induced cytopoenia (DIC), haemodynamic instability and higher costs.

Auxiliary heterotopic liver transplantation is applied with satisfactory results in some centres^3^. Intraportal hepatocyte transplantation has even been performed, but for selective indications^4^; its benefit in ALF has yet to be confirmed.

Artificial and bioartificial extracorporeal liver-support systems are still far from being incorporated into clinical routine; they should, however, be considered as a “bridge” while patients are waiting for transplants, which is the only definitive choice in most cases.
